# Europium (III) Organic Complexes in Porous Boron Nitride Microfibers: Efficient Hybrid Luminescent Material

**DOI:** 10.1038/srep34576

**Published:** 2016-09-30

**Authors:** Jing Lin, Congcong Feng, Xin He, Weijia Wang, Yi Fang, Zhenya Liu, Jie Li, Chengchun Tang, Yang Huang

**Affiliations:** 1 School of Materials Science and Engineering, Hebei University of Technology, Tianjin, 300130, P. R. China; 2 Hebei Key Laboratory of Boron Nitride Micro and Nano Materials, Hebei University of Technology, Tianjin, 300130, P. R. China

## Abstract

We report the design and synthesis of a novel kind of organic-inorganic hybrid material via the incorporation of europium (III) β-diketonate complexes (Eu(TTA)_3_, TTA = 2-thenoyltrifluoroacetone) into one-dimensional (1D) porous boron nitride (BN) microfibers. The developed Eu(TTA)_3_@BN hybrid composites with typical 1D fibrous morphology exhibit bright visible red-light emission on UV illumination. The confinement of Eu(TTA)_3_ within pores of BN microfibers not only decreases the aggregation-caused quenching in solid Eu(TTA)_3_, but also improves their thermal stabilities. Moreover, The strong interactions between Eu(TTA)_3_ and porous BN matrix result in an interesting energy transfer process from BN host to TTA ligand and TTA ligand to Eu^3+^ ions, leading to the remarkable increase of red emission. The synthetic approach should be a very promising strategy which can be easily expanded to other hybrid luminescent materials based on porous BN.

Researches on organic-inorganic hybrid materials have gathered immense attention because of their potential applications in various fields such as sensing, light-emitting, lithium ion battery, etc^1^,^2^,^3^,^4^,^5^. The combination of organic and inorganic components at a nanometer scale could allow us to generate unique properties in such complex systems. For example, encapsulating organic dyes into porous metal-organic frameworks (MOFs) can efficiently enhance their fluorescence quantum efficiency and tuning the emission color due to the confinement of dyes in the pores of MOFs^2^.

Trivalent lanthanide complexes with organic ligands are of great interest due to their outstanding luminescent properties. On one hand, lanthanide complexes could possess of characteristic and narrow emission bands with a full width at half maximum of less than 10 nm and long-lived excited states gaining from the luminescent centres of trivalent lanthanide ions (Ln^3+^). On the other hand, differing from the Ln^3+^ ions which have poor light absorption abilities due to the forbidden intraconfiguration *f-f* transition, lanthanide complexes exhibit sensitized emission because the organic ligands have large molar absorption coefficients and can effectively increase light absorption by “antenna effect”^6^,^7^. In spite of the outstanding luminescent properties of lanthanide complexes, the practical application of these complexes has still been limited due to their poor thermal stability and proccessibility^8^. To solve this problem, introducing lanthanide complexes in a stable porous matrix to form organic-inorganic hybrid materials has been intensively studied. So far, inorganic porous materials, such as zeolites^9^,^10^,^11^, mesoporous silica (SBA-15, MCM-41, etc)^12^,^13^,^14^,^15^,^16^,^17^,^18^ have been used as the matrix due to their special porous structure and thermal and chemical stabilities. The confinement of lanthanide complexes within porous matrix can not only improve their stabilities, but also decrease the aggregation-caused quenching in lanthanide complex, which would be beneficial for the effective luminescence^19^.

Porous boron nitride (BN) has attractive properties including low density, high thermal conductivity, superior oxidation resistance and chemical inertness. Particularly, the recently breakthrough on the preparation of porous BN with high specific surface areas and large pore volumes has gained this porous material much attention^20^,^21^,^22^,^23^,^24^,^25^,^26^,^27^,^28^. We envision that porous BN can be an ideal candidate as the matrix for lanthanide complexes. Notwithstanding the possibility, porous BN holding characteristic sp^2^-bonded honeycomb structure is quite different with the traditional oxide porous matrix from both the chemical and physical view of point. So-derived specific interactions with the lanthanide complexes will surely influence the luminescence performance of the constructed hybrid materials. Unfortunately, such lanthanide complex/porous BN hybrid luminescent materials have not been explored so far.

In this paper, we report the design of novel lanthanide complex/porous BN organic-inorganic hybrid materials via the incorporation of europium (III) β-diketonate complexes (Eu(TTA)_3_, TTA = 2-thenoyltrifluoroacetone) into porous BN microfibers. The Eu(TTA)_3_@BN composite possesses one-dimensional (1D) fibrous morphology similar with pure porous BN matrix. The emission intensity and lifetime of Eu^3+^ ions are significantly enhanced compared with pure europium complexes. The thermal stability of Eu(TTA)_3_ has also been improved due to the protection of porous BN matrix. Interestingly, due to the strong interactions between europium complexes and porous BN, efficient energy transfer from BN to TTA ligand and TTA ligand to Eu^3+^ ions takes place in the hybrid system, leading to the great enhancement of red emission. This novel organic-inorganic hybrid material is envisaged to become highly valuable in lighting devices and biomedical analysis.

## Results and Discussion

**Figure 1:**
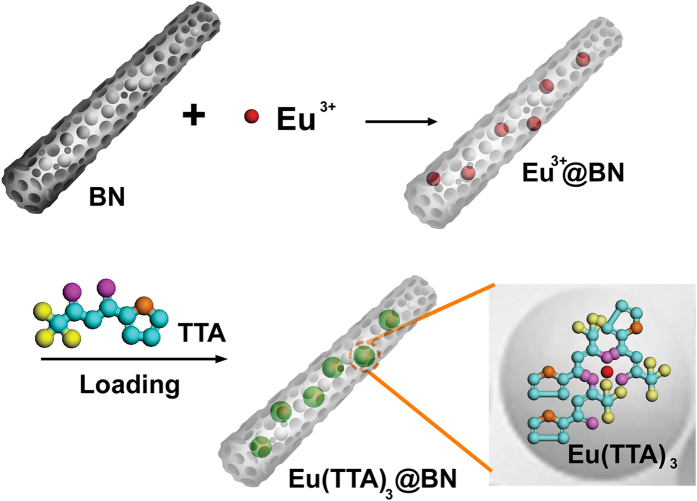
Illustration of the procedure for synthesis of Eu(TTA)_3_@BN hybrid microfibers.

Figure 1 illustrates the procedure for synthesis of the organic-inorganic hybrid material Eu(TTA)_3_@BN. The porous BN products used as the inorganic hosts are mainly some microfibers with main characteristic pore sizes of ~1.3 and ~3.9 nm. The specific surface area of the porous BN is ~1600 m^2^ g^−1^ and the pore volume is ~0.9 cm^3^ g^−1 ^23. The synthesis of Eu(TTA)_3_@BN was realized via a two-step method: the loading of Eu^3+^ ions into porous BN microfibers was achieved by adsorption of Eu^3+^ ions in ethanol solution dispersed with porous BN, and then the organic ligands TTA was inserted into Eu^3+^@BN by a gas diffusion method. Our previous studies indicate that the as-prepared porous BN exhibits excellent adsorption performance for various metal ions due to their high specific area, large pore volume and dipolar nature of B-N bonds. Especially, the surface of porous BN is negatively charged, so the metal ions can be adsorbed on porous BN by the electrostatic interactions^23^,^24^. After obtaining Eu^3+^@BN microfibers, the formation of organic-inorganic hybrid Eu(TTA)_3_@BN can be realized by coordination reaction between TTA and Eu^3+^ ions in porous BN microfibers. X-ray diffraction (XRD) result of hybrid product is quite similar to that of starting porous BN, indicating the amorphous phase of organic Eu(TTA)_3_ (Supporting Information, Fig. S1).

**Figure 2:**
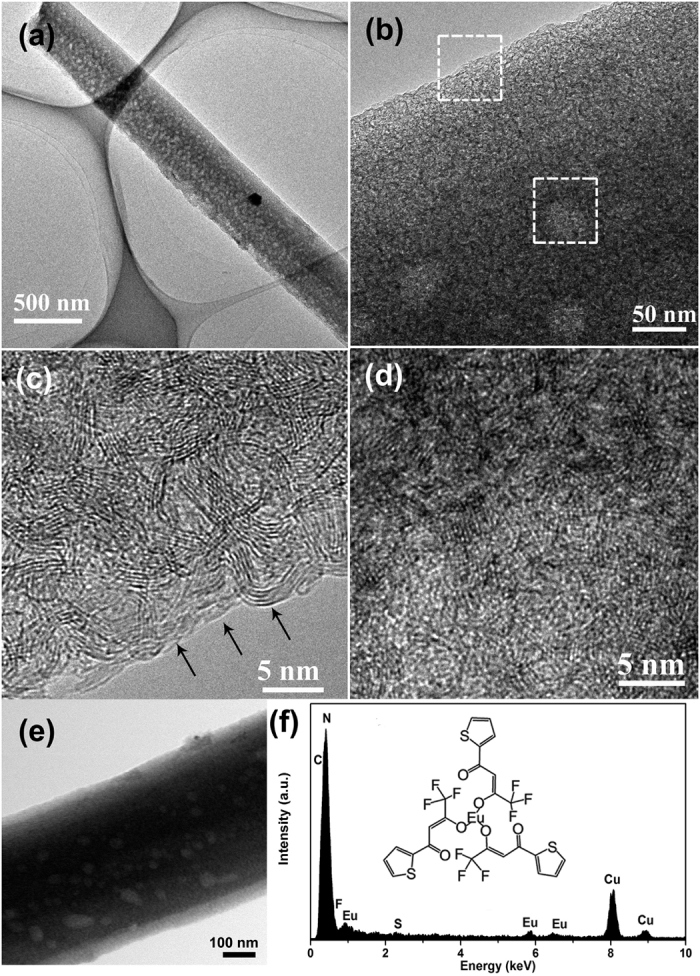
(**a**) Low-magnification TEM image of Eu(TTA)_3_@BN hybrid product, revealing the fibrous morphology; (**b**) Enlarged TEM image of the microfiber; (**c**, **d**) HRTEM images taken from the two areas labelled by the two dashed frames marked in (**b**), respectively; (**e**) STEM image and (**f**) the corresponding EDS spectrum taken from a single Eu(TTA)_3_@BN microfiber.

The microstructures and compositions of the Eu(TTA)_3_@BN hybrid product were further examined in transmission electron microscopy (TEM). A typical low-magnification TEM image (Fig. 2a) indicates that the product keeps fibrous morphology as as-prepared porous BN microfiber. The diameter of the microfiber here is ~0.5 μm. The bright spots on the microfiber shown in the enlarged TEM image (Fig. 2b) reveal some mesopores with the diameters of tens nm existing in the microfiber. Figure 2b also shows that the surface of the microfiber is quite clear. High resolution TEM image in Fig. 2c shows the edge of the microfiber. The crystallized BN layers (marked by the arrows) is very clear without any adhered substances. High resolution TEM image (Fig. 2d) taking from the centre of the microfibers shows an amorphous phase covered over the microfiber. The amorphous phase could be attributed to the Eu(TTA)_3_ on BN microfibers. To further confirm our success in introducing Eu(TTA)_3_, a composition analysis was carried out. Figure 2e,f show scanning transmission electron microscopy (STEM) image and the corresponding energy-dispersive X-ray spectroscopy (EDS) spectrum taken from a single Eu(TTA)_3_@BN microfiber. The peaks form C, F, Eu and S elements can be seen clearly. So we can confirm that the organic lanthanide complexes, Eu(TTA)_3_, have been successfully introduced into the BN microfibers. Besides, the specific surface area of Eu(TTA)_3_@BN microfibers has been measured as low as ~70 m^2^ g^−1^. Compared with pure porous BN microfibers which possess a very high specific surface area (~1600 m^2^ g^−1^), the significantly decrease of specific surface area implies that the lanthanide complexes has been encapsulated inside the pores of the BN. With a brief summary of the above results, we infer that the lanthanide complexes could be encapsulated inside the pore channels of BN microfiber, rather than coated on their outside surface.

**Figure 3:**
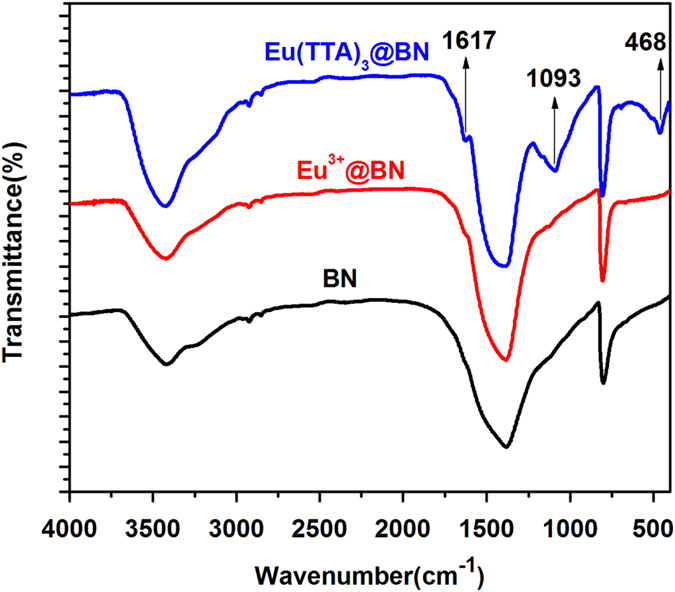
FTIR spectra of porous BN (**a**), Eu^3+^@BN (**b**) and Eu(TTA)_3_@BN (**c**) samples.

Fourier transform infrared (FTIR) analysis was utilized to study the loading of Eu(TTA)_3_ in porous BN. Figure 3 shows the FTIR spectrum of as-prepared Eu(TTA)_3_@BN sample. The spectra of pure porous BN and Eu^3+^@BN are also shown for comparison. The FTIR spectrum of porous BN shows peaks at ~1391 cm^−1^ and ~805 cm^−1^, corresponding to B-N stretching vibrations and B-N-B bending vibrations^29^, respectively. The peak at ~3426 cm^−1^ is attributed to the vibrations of B-OH/B-NH_2_ group^23^. In comparison, the FTIR spectrum of Eu^3+^@BN is quite similar to that of porous BN, although XPS analysis (Fig. S2, Supporting Information) clearly indicates that the Eu^3+^ has already been loaded. There is no noticeable shift for all the peaks from porous BN after incorporating of Eu^3+^, which means that the Eu^3+^ ions are adsorbed on BN by weak interactions, i.e. electrostatic interactions, rather than strong chemical bonding. After loading of Eu^3+^ and TTA on the porous BN successively, some new absorption peaks at ~1617 cm^−1^, ~1093 cm^−1^ and ~468 cm^−1^ can be observed. The peak at ~1617 cm^−1^ is assigned to the C=O stretching band which is related to the ligand (TTA); the peak at ~468 cm^−1^ could be attributed to the stretching Eu-O bond, indicating ligands are indeed coordinated with Eu^3+^ ions through the oxygen in the complex ligands; another peak observed at ~1093 cm^−1^ is corresponds to the B-N-O group, indicating that the strong interactions also occur between ligand TTA and BN host. The strong interaction between the europium complexes and porous BN is very import for avoiding the nonhomogeneous distribution and leakage of lanthanide complexes in the porous matrix.

**Figure 4:**
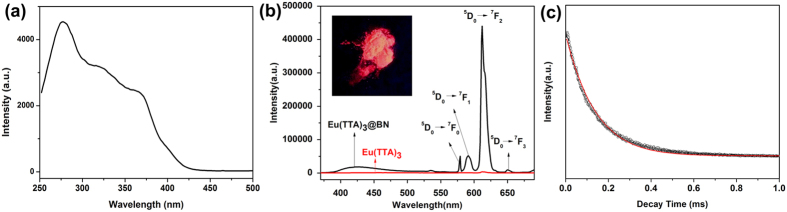
(**a**) Excitation spectrum of Eu(TTA)_3_@BN samples monitored at 615 nm. (**b**) Emission spectra of Eu(TTA)_3_@BN and solid Eu(TTA)_3_·nH_2_O samples excited by 280 nm. (inset) Photo image of Eu(TTA)_3_@BN excited by UV light, showing intense red light emission. (**c**) Decay curve of Eu(TTA)_3_@BN sample.

The excitation and emission spectra of Eu(TTA)_3_@BN microfibers were examined at room temperature. The excitation spectrum monitored at 615 nm displays a broad band ranging from 250 to 400 nm (Fig. 4a). The absence of sharp peaks corresponding to the *f-f* transitions of Eu^3+^ ions indicates that effective energy transfer takes place to Eu^3+^ ions^8^. Upon excited at 280 nm, the Eu(TTA)_3_@BN exhibits intense red emission with characteristic peaks of 581, 591, 615 and 653 nm, which correspond to the excited state ^5^D_0_ → ^7^F_J_ (J = 0–3) of Eu^3+^ ions, respectively (Fig. 4b). To make comparisons, we also measured the emission spectrum of pure Eu(TTA)_3_·nH_2_O in the solid state. As shown in Fig. 4b, very weak red emission can be observed in solid Eu(TTA)_3_·nH_2_O. The results demonstrate that after loading of Eu(TTA)_3_ in BN host, the nonradiative energy transfer process between Eu(TTA)_3_ can be successfully restrained, which would otherwise quench the red emission. The intense red light emission can be easily visible by eye upon 365 nm UV excitation, as shown in inset of Fig. 4b. The decay curve of Eu(TTA)_3_@BN under excitation of 280 nm is shown in Fig. 4c. The curve can be well fitted mono-exponentially and the value of lifetime τ is calculated to be 0.361 ms. This value is longer than lifetime of pure Eu(TTA)_3_·nH_2_O (τ = 0.21 ms, shown in Supporting Information, Fig. S3), which can be attributed to the inhibition of nonradiative deactivation pathways by confinement in the pores of BN host^8^.

**Figure 5:**
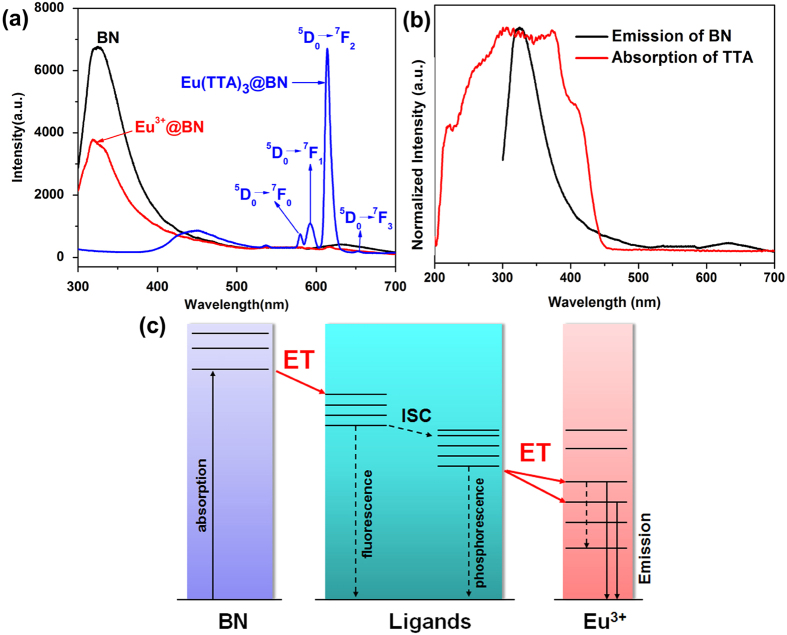
(**a**) Emission spectra of pure BN (black), Eu^3+^@BN (red) and Eu(TTA)_3_@BN (blue) samples. (**b**) Emission spectrum of pure BN (black) and UV-vis absorption spectrum of ligands TTA (red). (**c**) The schematic of the energy transfer process in Eu(TTA)_3_@BN system. ET = energy transfer, ISC = intersystem crossing.

In order to further clarify the reason for the great enhancement of red emission in the Eu(TTA)_3_@BN system, we compare the photoluminescence (PL) of pure BN, Eu^3+^@BN and Eu(TTA)_3_@BN samples, as shown in Fig. 5a. Pure porous BN microfibers exhibit strong UV emission centered at ~320 nm, which can be attributed to the defect-related centers or intrinsic impurities in BN^30^. After adsorption of Eu^3+^, the Eu^3+^@BN sample shows similar UV emission band and a very weak peak at ~613 nm, which corresponds to the red emission of Eu^3+^ ions. Since the *f-f* transitions of lanthanide ions are spin forbidden, it is hard to generate efficient luminescence emission by direct excitation. Through coordination to TTA ligands, the red emission intensity of Eu(TTA)_3_@BN is significantly enhanced, which can be attributed to the “antenna effect”^31^,^32^. Effective energy transfer from TTA ligands to Eu^3+^ ions takes place, resulting in sensitized red emission. Interestingly, we also find the UV emission of BN host disappears in Eu(TTA)_3_@BN, and a weak broad band centered at ~450 nm can be obtained. The ~450 nm band origins from the C- and/or O-related impurities in BN^33^,^34^. After coordination of TTA ligands with Eu^3+^ ions in BN host, the formation of B-N-O bonds makes it possible to obtain the weak blue emission from BN. Besides, the great suppressed UV emission indicates effective energy transfer from BN host to Eu(TTA)_3_ may be occurred. As shown in Fig. 5b, the absorption spectrum of TTA shows a broad band between 200 and 450 nm, while the pure BN host exhibits strong UV emission centered at ~320 nm. The spectral overlap between the emission of BN host and absorption of TTA results in an efficient BN-to-TTA energy transfer behavior. PL maps of BN host and Eu(TTA)_3_ also indicate similar energy transfer results (Supporting Information, Fig. S4). We believe the energy transfer process in the Eu(TTA)_3_@BN system can be described as follows (Fig. 5c)^6^: (1) the BN host is excited from the ground state to the excited state by absorbing UV energy; (2) the energy of the BN excited state is transferred to the ligands TTA; (3) the ligands TTA is excited to the singlet excited state, and then the energy is transferred to the triplet excited state through intersystem crossing (ISC); (4) energy transfers from the triplet state of the ligands to the excited 4*f* states of Eu^3+^ ions; (5) red emission can be obtained through the *f-f* transitions in Eu^3+^ ions. This interesting energy transfer process is quite different from those in earlier reported hybrid system, i.e. lanthanide complexes encapsulated in zeolites, SBA-15, MCM-41, etc, in which only effective energy transfer from the organic ligands to the Ln^3+^ ion (the so-called “antenna effects”) takes place. Herein our results represent that when the Eu(TTA)_3_@BN hybrid system is excited at 280 nm, efficient energy transfer of BN-to-TTA-to-Eu^3+^ occurs, leading to the great enhancement of red emission.

We have also studied the PL properties of Eu(TTA)_3_@BN samples with different Eu concentrations, as shown in Fig. S5, Supporting Information. All of the emission spectra consist of similar weak broad blue band emission and intense red emission peaks. With an increase of Eu contents from 0.00005 mol/L to 0.005 mol/L, the intensity of blue band decreases gradually, while the intensity of red emission peaks increases significantly. However, when the Eu contents increases to 0.05 mol/L, a great decrease of red emission has been observed. The PL quenching may result from the nonradiative energy transfer process between Eu(TTA)_3_ in a high concentration.

**Figure 6:**
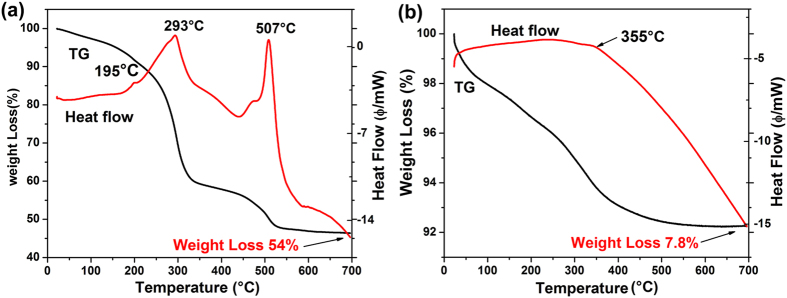
TG-DTA curves of (**a**) Eu(TTA)_3_·nH_2_O and (**b**) Eu(TTA)_3_@BN, in the presence of air.

The thermal stability of Eu(TTA)_3_@BN sample was studied by thermogravimetric (TG) analysis in the presence of air. Besides, the thermal stability of pure Eu(TTA)_3_·nH_2_O was also studied for comparison. As shown in Fig. 6a, the curve of pure Eu(TTA)_3_·nH_2_O complex shows a weight loss of ~54% for the entire process. The first weight loss peak at 195 °C corresponds to the loss of coordinate water, indicating the decomposition of the complexes starts. The weight loss occurs between 230 °C and 550 °C can be attribute to the decomposition of Eu(TTA)_3_. In detail, the second peak at 293 °C is related to the decomposition of the three ligands in the complex, while the third peak appears at 507 °C is due to the oxidation of the complex and finally Eu_2_O_3_ could be generated^35^. Figure 6b shows the TG curve of Eu(TTA)_3_@BN, displaying a weight loss of only ~7.8% for the entire process. The first fast weight loss step starts from room temperature and ends at about 50 °C, with a weight loss of ~2%, is related to the remaining gas desorption from the sample. The second step shows a weight loss peak at 355 °C due to the decomposition of Eu(TTA)_3_. The comparison indicates that the Eu(TTA)_3_@BN hybrid material displays greatly enhanced thermal stability compared to the pure Eu(TTA)_3_·nH_2_O complex. We believe that the space restriction of the europium (III) complexes by the pores of BN microfibers is the main factor for the improvement of their thermal stability.

## Conclusions

We designed and synthesized a novel kind of organic-inorganic hybrid materials via the incorporation of europium (III) complexes into 1D porous BN microfibers. TEM analysis demonstrated the amorphous europium (III) complexes were encapsulated inside porous BN, forming 1D Eu(TTA)_3_@BN hybrid composites. The developed Eu(TTA)_3_@BN hybrid composites showed bright visible red-light emission on UV illumination. Our results demonstrated an interesting and efficient energy transfer from BN host to TTA ligand and TTA ligand to Eu^3+^ ions occurred during the photoluminescence process, which was quite different from earlier reports regarding lanthanide complexes encapsulated within other porous matrix. The confinement of the europium (III) complexes within the pores of BN microfibers not only improved their thermal stabilities, but also decreased the aggregation-caused quenching in solid europium (III) complexes, leading to the remarkable increase of red emission. The designed synthetic approach we report here offers a great flexibility in rational design of many other hybrid luminescent materials based on porous BN.

## Experimental Section

### Staring materials

Porous BN microfibers were synthesized in relation to the reported procedure using H_3_BO_3_ and C_3_N_6_H_6_ as the starting materials^23^. 2-Thenoyltrifluroacetone (99%, TTA, Aldrich) was used as standard. Eu(TTA)_3_·nH_2_O was produced by the reported method^35^. All the other reagents are analytical pure.

### Preparation of Eu^3+^@BN microfibers

Firstly, loading of Eu^3+^ ions into porous BN microfibers was achieved by adsorption method. Briefly, 1 g of porous BN microfibers were dispersed in 1 L of ethanol to obtain a BN suspension. Then Eu(NO_3_)_3_·6H_2_O was dissolved in ethanol with a desired concentration (0.0005 mol/L), and added dropwise into the BN suspension. Then the mixed solution was stirred for 12 h and filtered. After drying at 80 °C in air overnight, white powder was obtained.

### Synthesis of Eu(TTA)_3_@BN microfibers

Eu(TTA)_3_@BN microfibers were prepared by a gas diffusion method. In detail, 0.42 g of the as-prepared Eu^3+^@BN white powder and 0.33 g of TTA were grinded together and then put into a gas diffusion flack. The flack was set inside of oil bath pan at 60 °C for 1 h under a vacuum condition. Then the temperature was raised up to 100 °C and kept for 24 h. Finally, the products were washed with acetonitrile and dried in air at 80 °C, respectively.

### Characterization

The structures of the as-prepared samples were characterized using an X-ray powder diffraction (XRD, BRUKER D8 FOCUS). The chemical bonding state of the products and the surface functional groups was examined by a Fourier transform infrared spectrophotometer (FTIR) (VECTOR22). The microstructures and compositions of the products were analysed using a transmission electron microscope (TEM, Philips Tecnai F20) equipped with an energy-dispersive X-ray spectrometer (EDS). For this, the powder samples were dispersed in ethanol, and then dripped dropwise onto the copper meshes covered with holey carbon films. The luminescent properties of the products were measured on the fluorescence spectrophotometer (F-7000 and FL3-22) and thermal stability analysis was carried out using a thermogravimetric analyzer (TG) (SDT Q-600) under normal atmosphere with stream of (100 mL min^−1^), at a heating rate of 5 °C min^−1^.

## Additional Information

**How to cite this article**: Lin, J. *et al*. Europium (III) Organic Complexes in Porous Boron Nitride Microfibers: Efficient Hybrid Luminescent Material. *Sci. Rep.* **6**, 34576; doi: 10.1038/srep34576 (2016).

## Supplementary Material

Supplementary Information
