# Supplementary material for: Europium (III) Organic Complexes in Porous Boron Nitride Microfibers: Efficient Hybrid Luminescent Material
Source: Sci Rep. 2016 Sep 30;6:34576. doi: 10.1038/srep34576 (PMC5043355; doi:10.1038/srep34576)
Supplement: Supplementary Information [file srep34576-s1.doc]

**Supporting Information**

**Europium (III) Organic Complexes in Porous Boron Nitride Microfibers: Efficient Hybrid Luminescent Material**

Jing Lin^1,2,*^, Congcong Feng^1,2^, Xin He^1,2^, Weijia Wang^1,2^, Yi Fang^1,2^, Zhenya Liu^1,2^, Jie Li^1,2^, Chengchun Tang^1,2^, Yang Huang^1,2,*^

^1^School of Materials Science and Engineering, Hebei University of Technology, Tianjin 300130, P. R. China

^2^ Hebei Key Laboratory of Boron Nitride Micro and Nano Materials, Hebei University of Technology, Tianjin 300130, P. R. China

* Corresponding author. E-mail: linjing@hebut.edu.cn (J. L.); [huangyang@hebut.edu.cn](mailto:huangyang@hebut.edu.cn) (Y. H.)


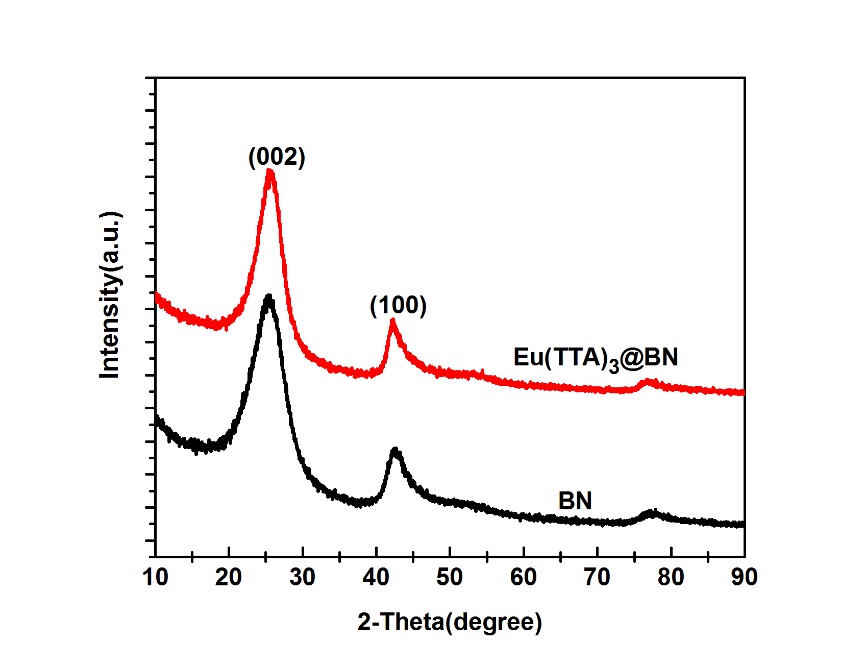


**Figure S1** XRD patterns of porous BN (black) and Eu(TTA)_3_@BN (red) samples.


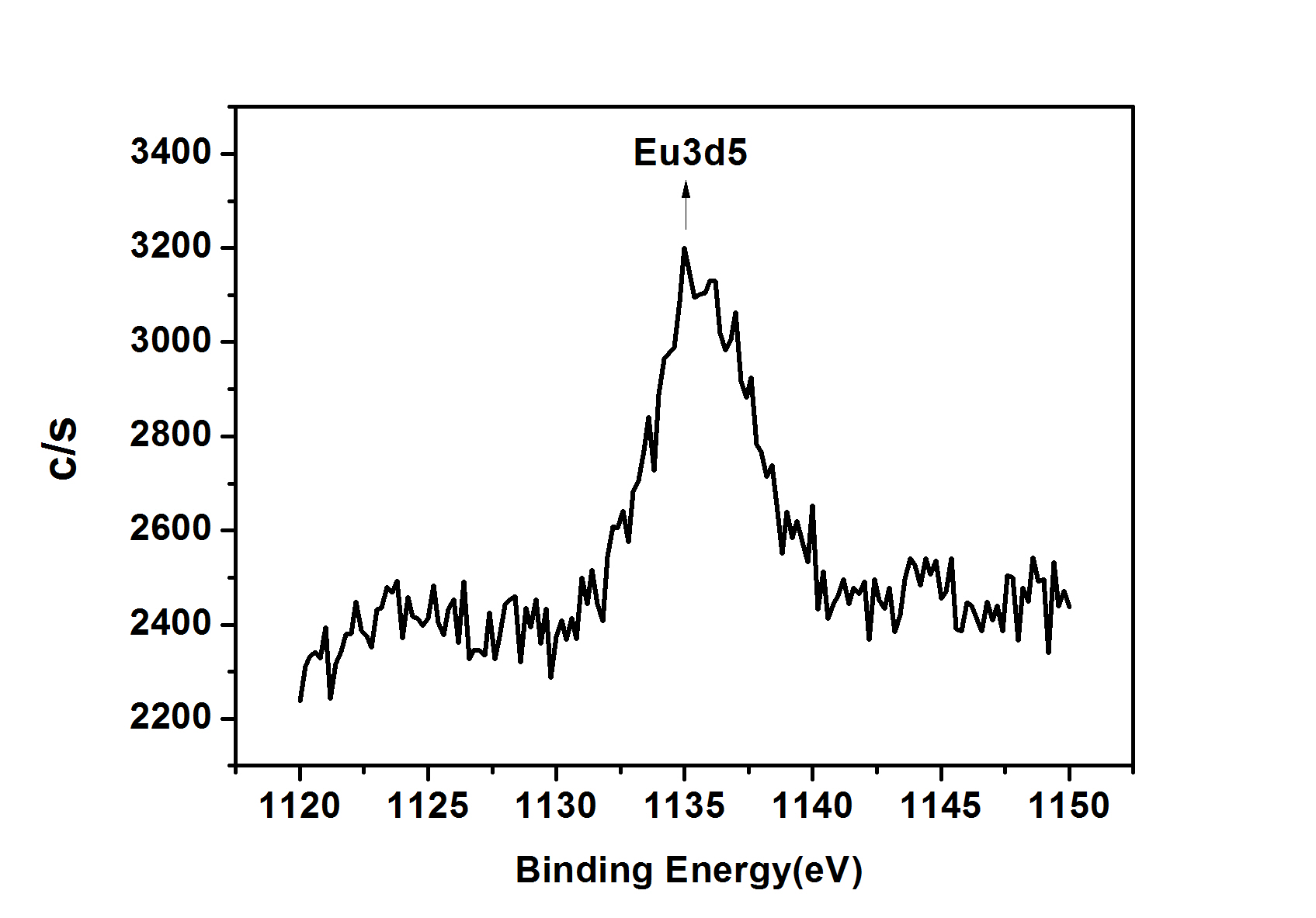


**Figure S2** XPS spectrum of Eu 3d in Eu@BN sample. The binding energy of Eu 3d_5/2_ is found to be 1135.3 eV, which corresponds to the characteristic feature of Eu^3+^.


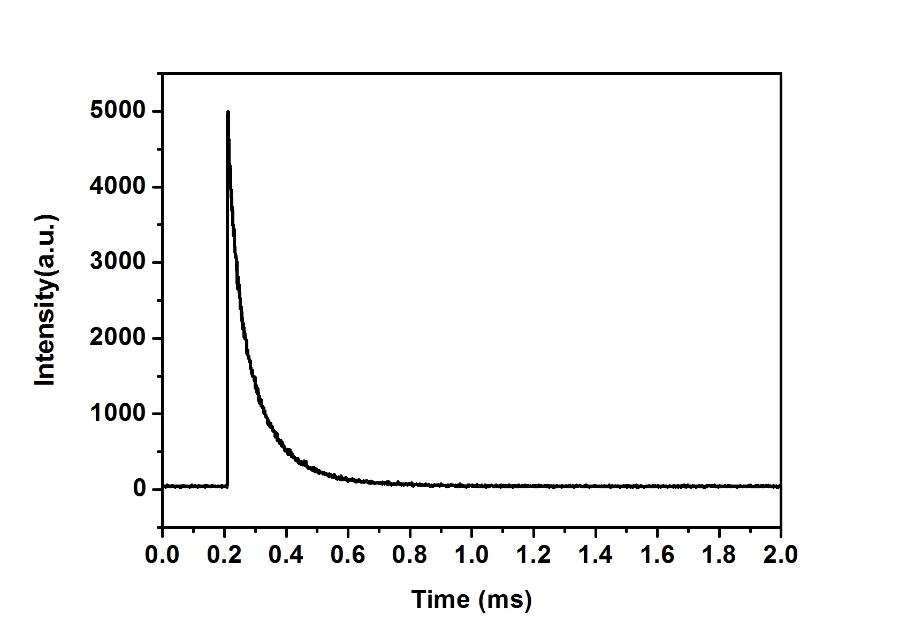


**Figure S3** Decay curve of pure Eu(TTA)_3_·nH_2_O sample. The curve can be well fitted by the bi-exponential function as the formula *I*(t) = *I*_1_ exp(*-t/τ*_1_) + *I*_2_ exp(*-t/τ*_2_). The value of lifetime *τ* is calculated to be 0.21 ms.


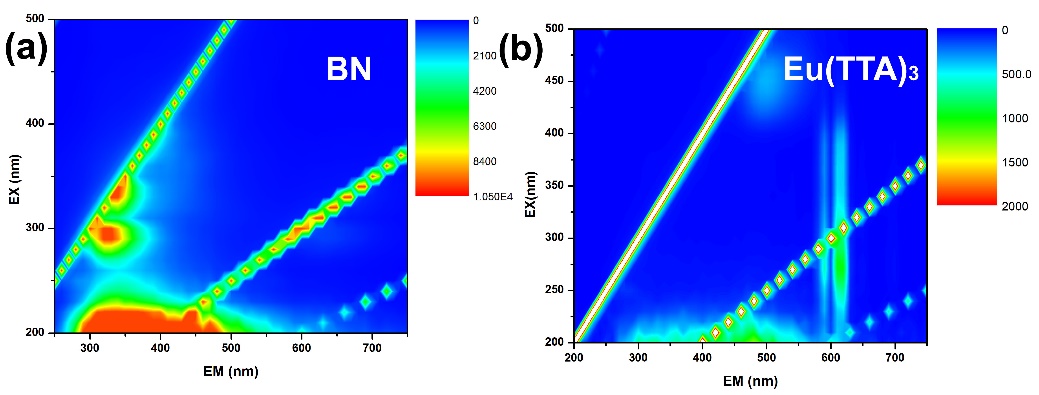


**Figure S4** PL maps of porous BN (a) and Eu(TTA)_3_ (b). It clearly indicates that the Eu(TTA)_3_ can be excited by UV light with wavelength range from 200 to 400 nm, while the pure BN host exhibits strong UV emission at ~320 nm (also shown in Figure 5a). The spectral overlap between the emission of BN host and excitation of Eu(TTA)_3_ results in an efficient BN-to-Eu(TTA)_3_ energy transfer behavior.

**
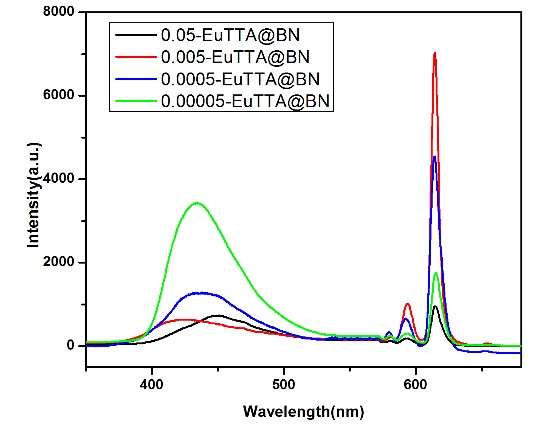
**

**Figure S5** Emission spectra of Eu(TTA)_3_@BN samples with different Eu concentrations. The samples were prepared by changing the concentrations of Eu(NO_3_)_3_·6H_2_O in ethanol solution (0.00005 mol/L, 0.0005 mol/L, 0.005 mol/L, and 0.05 mol/L). All of the emission spectra consist of similar weak broad blue band emission and intense red emission peaks. The ~450 nm band origins from the C- and/or O-related impurities in BN, while the sharp red emission peaks centered at 581, 591, 615 and 653 nm correspond to the excited state ^5^D_0_→^7^F_J_ (J=0–3) of Eu^3+^ ions, respectively. With an increase of Eu contents from 0.00005 mol/L to 0.0005 mol/L to 0.005 mol/L, the intensity of blue band decreases gradually, while the intensity of red emission peaks increases significantly. However, when the Eu contents increase to 0.05 mol/L, a great decrease of red emission has been observed. The photoluminescence quenching may result from the nonradiative energy transfer process between Eu(TTA)_3_ in a high concentration.
